# Is it effective to do mathematical analysis for the etiology of nocturia using the nocturia indices derived from the frequency volume chart?: A retrospective observational study

**DOI:** 10.1097/MD.0000000000042222

**Published:** 2025-05-09

**Authors:** Sungchan Park, Woocheol Kang, Dong-Gi Lee, Hoyoung Bae, Eun Ji Park, Ji Hyung Yoon, Taekmin Kwon, Kyung Hyun Moon, Sang Hyeon Cheon, Seong Cheol Kim

**Affiliations:** a Department of Urology, Ulsan University Hospital, University of Ulsan College of Medicine, Ulsan, Korea; b Basic-Clinical Convergence Research Institute, University of Ulsan, Ulsan, Korea; c Department of Urology, Kyung Hee University Hospital at Gangdong, College of Medicine, Kyung Hee University, Seoul, Korea; d BigData Center, Ulsan University Hospital, Ulsan, Korea.

**Keywords:** decreased bladder capacity, frequency volume chart, mathematical calculation, maximal voided volume, nocturia

## Abstract

This study aims to determine the problems involved in using a mathematical calculation to analyze the pathophysiology of nocturia using a frequency volume chart (FVC). In this retrospective study, we reviewed 253 patients who completed the FVC during ≥ 3 days for nocturia from January 2017 to February 2019. The etiology of nocturia was defined as a combination of 4 pathophysiologies, including nocturnal polyuria (NP), decreased bladder capacity (dBC), decreased nocturnal bladder capacity, and global polyuria. To analyze the differences according to diagnostic criteria for NP and dBC, 2 definitions were classified. Definition 1: NP is NP index (NPi) > 0.35 and dBC as maximal voided volume (MVV) < 325 mL. Definition 2: NP is NPi = 0.20 to 0.33 depending on age and dBC as MVV < 200 mL. The MVV in the FVC for ≥3 days was significantly higher than that for 1 day (330 mL vs 400 mL, *P* < .001). During the entire FVC period, all pathophysiology remained unchanged in only 16.6% of cases (inter-day variation). Of 323 days in which nocturia occurred ≥ 2 per night, 118 days (36.5%) full bladder voiding of nocturia was not the same for each day (inter-nocturia variation). According to definitions 1 and 2, the serial changes of NP and dBC showed different patterns. The mathematical calculation used in analyzing the pathophysiology of nocturia using an FVC has problems such as differences according to the duration of FVC and inability to express inter-day and inter-nocturia variations. Therefore, this mathematical calculation is not helpful when determining the treatment modality for nocturia.

## 1. Introduction

Nocturia is the most bothersome symptom among lower urinary tract symptoms, and it is also the least responsive symptom to medication.^[1]^ This least responsiveness may be due to the wrong treatment method for nocturia thus far, and a more accurate diagnosis for nocturia is needed to make up for this. A frequency volume chart (FVC) is very useful to determine the pathophysiology of nocturia.^[2,3]^ The FVC is noninvasive, and more than 80% of patients can complete it accurately for ≥2 days.^[4]^ The FVC can be used for both adults and children, and it is also possible to evaluate bladder capacity and response after drug medication.^[5,6]^

To date, mathematical calculations are used to analyze the pathophysiology of nocturia using the FVC.^[7–10]^ It expresses the average etiology of nocturia during the FVC writing period. Therefore, it assumes that all nocturia occurring during the FVC writing period has the same etiology. If the above assumption is not correct, the mathematical calculation to analyze the etiology of nocturia cannot be effective.

Therefore, this study aimed to determine what problems exist when using a mathematical calculation to analyze the pathophysiology of nocturia using the FVC.

Since the mathematical calculation used to analyze the etiology of nocturia using the FVC represents the average value during ≥3 days, we hypothesized that there is a limit in determining the appropriate treatment for nocturia.

## 2. Materials and methods

### 2.1. Study design

This was a retrospective observational study approved by the Institutional Review Board of Haeundae Paik Hospital (IRB approval number: 2019-11-040 and 2019-11-041); it conformed to the principles outlined in the Declaration of Helsinki. The requirement for informed consent was waived by the Institutional Review Board of Haeundae Paik Hospital given the retrospective study design and the anonymization of the data included in the study. This study was conducted based on a previously reported cohort.^[11]^ We reviewed 311 patients who visited our hospital with complaints of nocturia and underwent FVC from January 2017 to February 2019. The inclusion criterion was completion of FVC for ≥24 hours (the 24 hour-period beginning with the first-morning void and ending with the first-morning void of the following day). Exclusion criteria were the absence of nocturia on FVC, ≤24 hours of FVC, and completing FVC during < 3 days. We evaluated the age, sex, FVC (sleep and waking time, voided volume, voided time, maximal voided volume [MVV], actual nocturnal voids [ANV], 24-hour voided volume [24hV], nocturnal urine volume [NUV]).

### 2.2. Study definitions

The 24hV and NUV were defined according to International Continence Society terminology.^[12]^ MVV was defined as the largest voided volume recorded in each FVC. From the FVC variables, we derived the nocturia index (Ni; NUV/MVV), nocturnal polyuria (NP) index (NPi; NUV/24hV), predicted nocturnal void (PNV; Ni-1), and nocturnal bladder capacity index (NBCi; ANV-PNV). In this study, the etiology of nocturia was defined as a combination of 4 pathophysiologies, including NP, decreased bladder capacity (dBC), decreased nocturnal bladder capacity (dNBC), and global polyuria (GP). As reported in previous study, the NP and GP were defined as NPi > 0.35 regardless of age and 24hV > 2.5 L/d, respectively.^[11]^ The dBC was when the MVV was <325 mL, while dNBC was defined as NBCi > 0.

To analyze the inter-nocturnal variation, the full bladder voiding in nocturia was defined as a case in which voiding volume in nocturia was >350 mL when MVV during daytime (MVVDT) was ≥325 mL and greater than MVVDT when MVVDT was <325 mL.

To analyze the differences according to diagnostic criteria for NP and dBC, 2 definitions were classified. Definition 1 included NP as NPi > 0.35 and dBC as MVV < 325 mL. Definition 2 included NP as NPi 0.20 to 0.33 depending on age and dBC as MVV < 200 mL.

### 2.3. Analyses

Patient characteristics are presented as frequencies and percentages for categorical variables or as median and interquartile range (IQR) for continuous variables. The etiology of nocturia from 1-day FVC and ≥ 3 days was expressed using a Venn diagram of 4 risk factors (NP, dBC, dNBC, and GP). We used the linear-by-linear association test for the assessment of trends according to the number of nocturia episodes and age groups. The chi-square test was used to evaluate differences in the NP and dBC between the 2 definitions. Statistical significance was set at *P* < .05. SPSS version 25.0.0 (IBM Corp., Armonk) was used for statistical analysis.

## 3. Results

### 3.1. Participants

**Figure 1.**
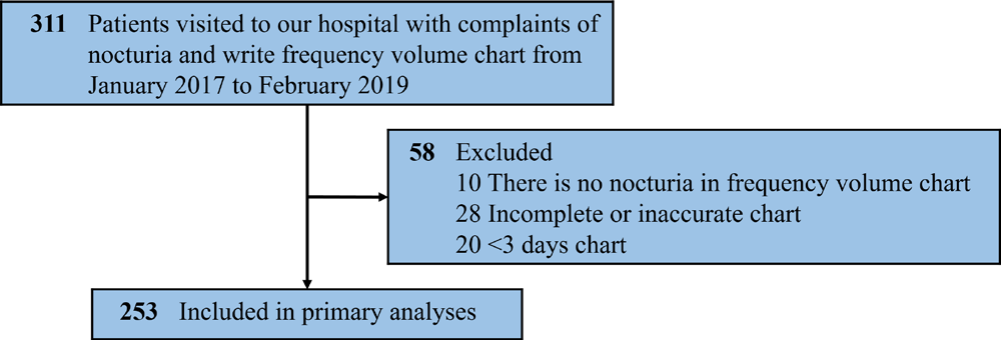
Overview of the study population.

A total of 311 patients were enrolled, of which 58 were excluded because 10 had no nocturia on FVC, 28 had incomplete or inaccurate FVC, and 20 had <3 days FVC (Fig. 1). The remaining 253 patients were included in the primary analyses. The median age was 64 years (IQR 59–72) and 56.5% of the participants were male. Of the 765 days from 253 FVCs, the median number of nocturia episodes was 1 per day (IQR 1–2); 45.9% had 1, 27.7% had 2, 9.9% had 3, and 4.6% had 4 or more nocturia episodes per day. Table 1 shows details of the participants and their FVC characteristics. The median 24hV was 1,780 mL (IQR 1,355–2,250) and the median NUV was 600 mL (IQR 430–850).

**Table 1:** Participant characteristics.

Variables	
Age (yr)	
Median	64
Range	23 to 84
IQR	59 to 72
Age group	
≤30s	11 (4.3%)
40s	19 (7.5%)
50s	51 (20.2%)
60s	96 (37.9%)
70s	59 (23.3%)
≥80s	17 (6.7%)
Sex (n, %)	
Male	143 (56.5%)
Female	110 (43.5%)
Number of nocturia episodes (per night)	1.5 ± 1.1
Median	1
Range	0 to 7
IQR	1 to 2
Sleep duration (min)	
Median	430
Range	130–745
IQR	380 to 490
24-h voided volume (mL)	
Median	1,780
Range	135–5,550
IQR	1,355–2,250
Nocturnal urine volume (mL)	
Median	600
Range	45 to 2,400
IQR	430 to 820
Maximal voided volume during 1 d (mL)	
Median	330
Range	30 to 1,600
IQR	300 to 450
Maximal voided volume during whole days (mL)	
Median	400
Range	40 to 1,600
IQR	300 to 500

Abbreviation: IQR = interquartile range.

### 3.2. Difference between 1 day and ≥3 days FVC

**Figure 2.**
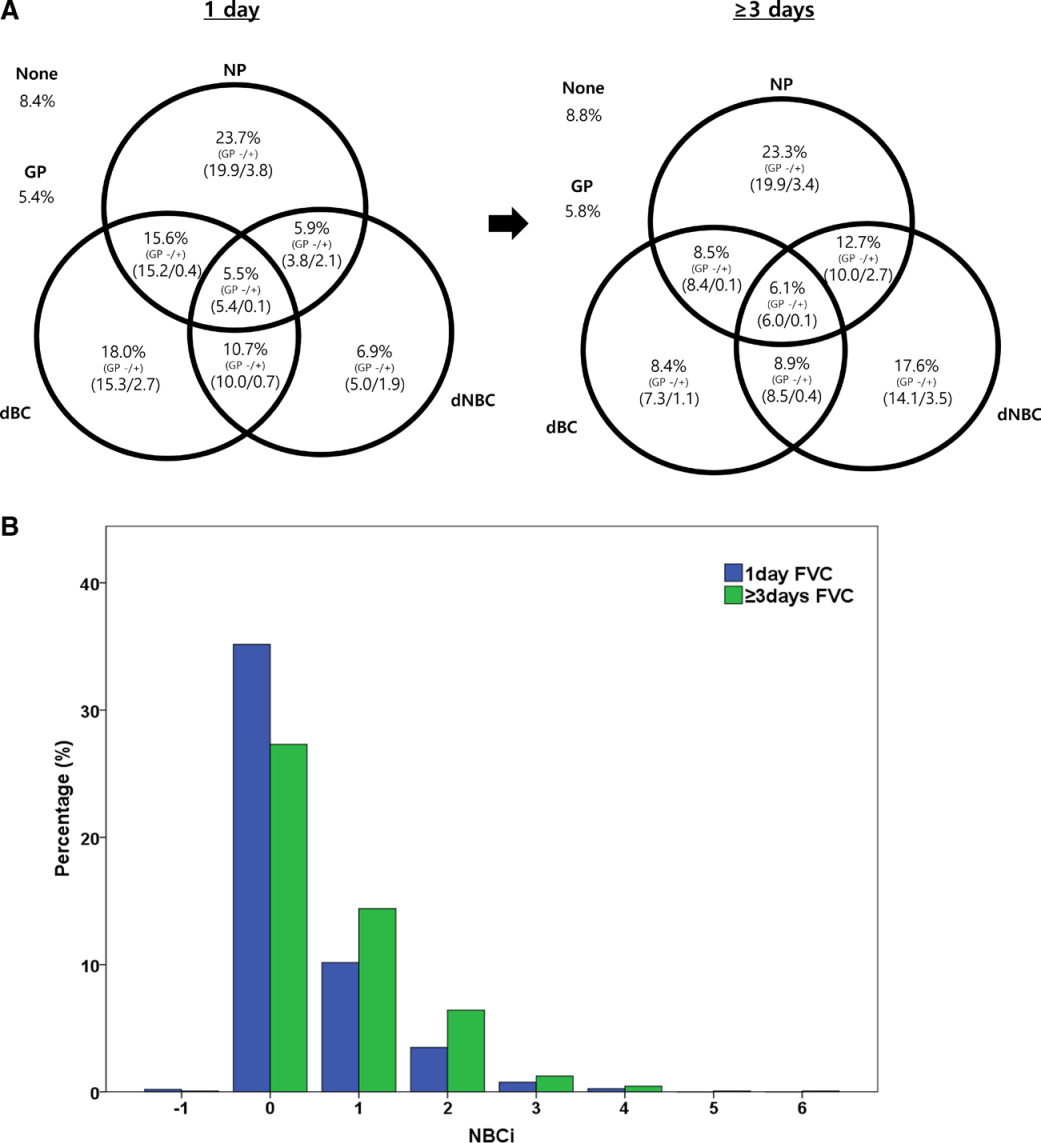
Difference between 1 d and ≥3 d. (a) Venn diagram showing the change of the etiology from 1-d FVC to ≥3 d FVC, (b) Ratio of values of NBCi in the FVC during 1 d and ≥3 d. dBC = decreased bladder capacity, dNBC = decreased nocturnal bladder capacity, FVC = frequency volume chart, GP = global polyuria, NBCi = nocturnal bladder capacity index, NP = nocturnal polyuria.

In the same participant’s FVC, the MVV in the FVC for the entire period was significantly higher than that of the 1-day FVC (330 mL vs 400 mL, *P* < .001; Fig. 2A and Appendix Table S1, Supplemental Digital Content, https://links.lww.com/MD/O778). Due to this change, the ratio of dBC in the pathophysiology diagnosed in the FVC of the entire period rather than the 1-day FVC decreased from 49.8% to 31.9% (*P* < .001); however, the ratio of dNBC increased from 29.0% to 45.3% (*P* < .001). When analyzed with the FVC for the entire period rather than the 1 day, 0 decreased but 1 or more increased in the NBCi value (Fig. 2B, *P* < .001).

### 3.3. Inter-day and inter-nocturia variations

**Figure 3.**
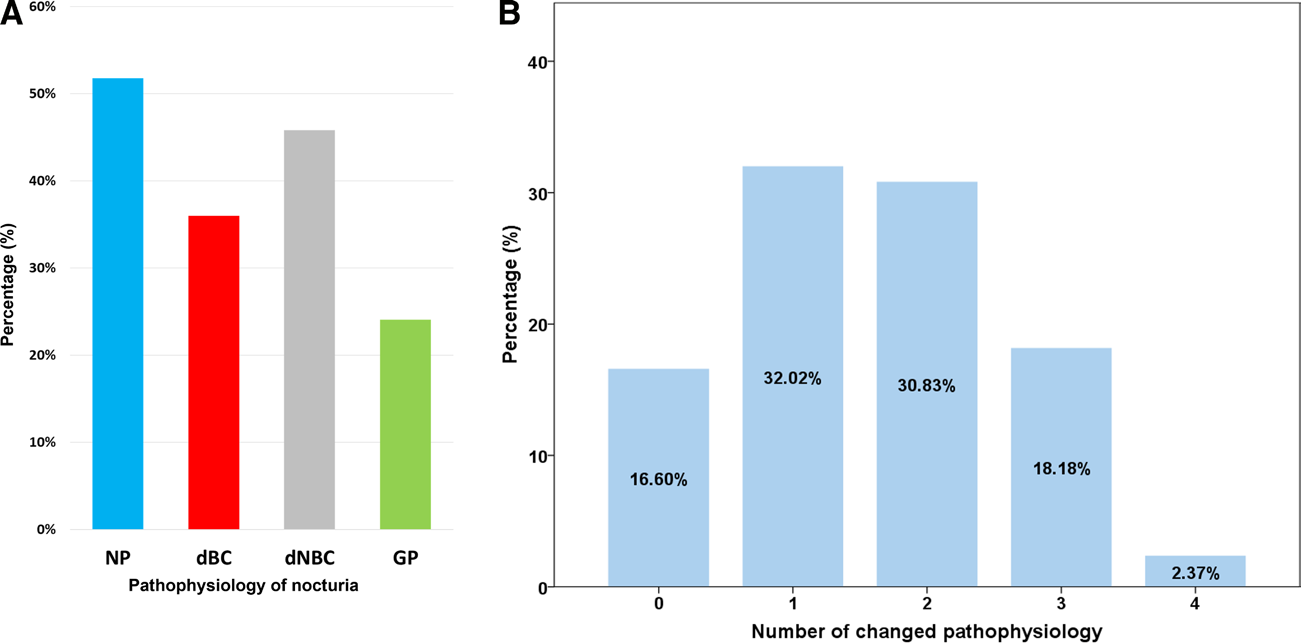
Inter-day variation. (A) Rate of change of the pathophysiology of nocturia diagnosed in each day during the entire period of FVC, (B) Number of the pathophysiology of nocturia changed during the entire period of FVC. dBC = decreased bladder capacity, dNBC = decreased nocturnal bladder capacity, FVC = frequency volume chart, GP = global polyuria, NP = nocturnal polyuria.

Diagnosis of NP, dBC, dNBC, and GP, which are the pathophysiology of nocturia, was changed in 51.8%, 36.0%, 45.8%, and 24.1%, respectively, during the entire period of the FVC (Fig. 3A). During the entire FVC period, all pathophysiology remained unchanged in only 16.6% of cases, but ≥ 2 was changed in 51.4% of cases (Fig. 3B). Among the 323 days in which nocturia occurred ≥ 2 per night, 118 days (36.5%) were the cases in which full bladder voiding of nocturia occurred on each day was not the same.

### 3.4. Difference according to diagnostic criteria for NP and dBC

**Figure 4.**
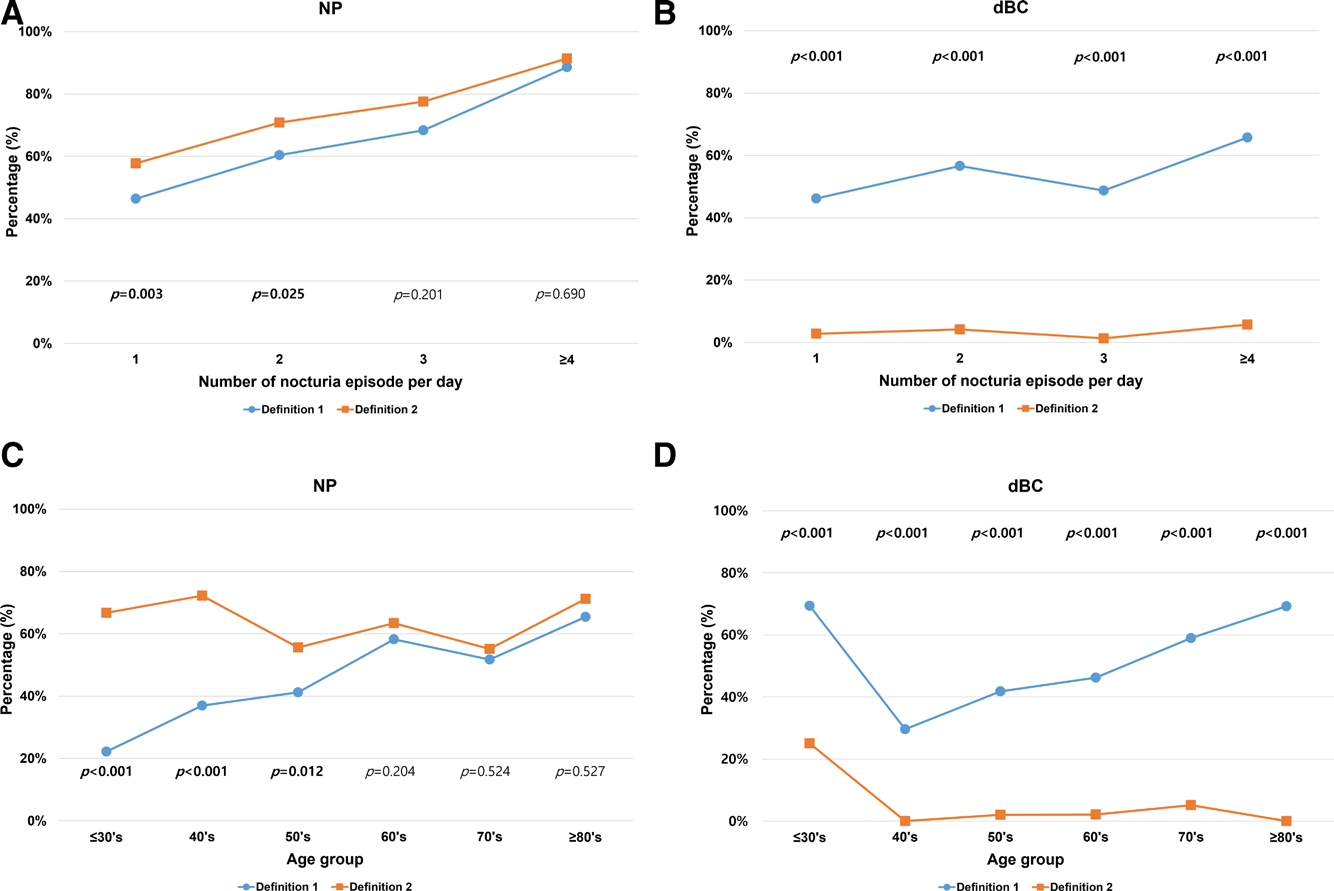
Difference according to diagnostic criteria for NP and dBC. Serial changes of 2 pathophysiology (NP and dBC) affecting nocturia according to the number of nocturia episodes (A and B) and age (C and D). Definition 1 (NP: NPi > 0.35 and dBC: MVV < 325 mL), definition 2 (NP: NPi0.20 to 0.33 and dBC: MVV < 200 mL). dBC = decreased bladder capacity, MVV = maximal voided volume, NP = nocturnal polyuria, NPi = nocturnal polyuria index.

NP was diagnosed more as the pathophysiology of nocturia in definition 2 than in definition 1 (61.2% vs 50.6%, *P* < .001). In both definitions, the NP as the pathophysiology of nocturia also tended to increase when the number of nocturia episodes increased (Fig. 4A). As the number of nocturia episodes increased in definition 1, the dBC showed a tendency to increase as the pathophysiology of nocturia, but it was not in definition 2 (Fig. 4A). Definition 1 showed a trend of increasing frequency of NP (Fig. 4C) and dBC (Fig. 4D) as the pathophysiology of nocturia with age; however, definition 2 did not show this trend.

## 4. Discussion

For the diagnosis of nocturia, the FVC is very important.^[2,3]^ This is because the pathophysiology (NP, dBC, dNBC, and GP) of nocturia can be determined by calculating the index related to nocturia through the FVC.^[11]^ However, the pathophysiology identified in this way is only risk factors and is not the cause of nocturia. In other words, it means that the possibility of nocturia is high if each risk factor is present, but it is able to be concluded that these are the direct causes of nocturia. In this paper, we explain 4 reasons why deriving pathophysiology using the FVC is not helpful in determining treatment methods for nocturia.

First, results could be analyzed differently depending on whether an FVC is completed for 1 day or 3 days. An important difference between a 1-day FVC and an FVC during ≥ 3 days is the MVV. As the number of days completing the FVC increases, the opportunity for a larger bladder capacity to be written could increase, which in turn could increase the MVV. This increase in the MVV reduced the diagnostic rate of dBC and acted as a factor in increasing the diagnostic rate of dNBC (NBCi = ANV-[NUV/MVV-1]). As described above, even with the same patient’s FVC, the etiology of nocturia diagnosed from the FVC could vary depending on the duration of the FVC.

Second, each day of nocturia did not always have the same etiology, and this point could not be expressed in the mathematical calculation using the FVC. In this paper, this was expressed as an inter-day variation. This paper showed that the etiology of nocturia on all days was the same in only 16.6%. Therefore, it is inappropriate to assume that the etiology of nocturia on each day is the same in the FVC completed for more than 2 days. In particular, for NP and dNBC as the pathophysiology of nocturia, approximately half of the cases changed during 3 days. There were several possible reasons for this change. First, when the value of each factor was close to the criteria cutoff of each diagnosis for the pathophysiology of nocturia, the diagnosis could be changed even with a small variation. Second, the daily living environment could not be considered the same. For example, if weekdays and weekends were included, the etiology of nocturia could be changed. Third, the etiology of nocturia could be changed as lifestyle was modified while writing the FVC.^[13]^ Regarding these reasons, the etiology of nocturia derived from the mathematical calculation based on the FVC cannot represent the etiology of nocturia for each day.

Third, whole nocturia occurring on the same day could not always be regarded as the same etiology, and this was unable to be expressed in the mathematical calculation using the FVC. In this paper, this was expressed as an inter-nocturia variation. To date, there was no way to objectively express the etiology of each nocturia. Therefore, in this study, the inter-nocturnal variation was evaluated by simply using whether nocturia occurs in the full bladder. This study showed that nocturia occurring on the same day was not the same as whether nocturia occurred in the full bladder in ≥ 35% of cases. It was found that all nocturia occurring on the same day did not occur due to the same etiology. Therefore, the etiology of each nocturia per day could not be expressed by the mathematical calculation based on the FVC.

Fourth, different results were obtained depending on how the diagnostic criteria for defining the pathophysiology of nocturia were established. There are 2 methods for determining the diagnostic criteria, which are absolute criteria and age or body weight-adjusted criteria.^[12,14]^ This study showed differences between absolute criteria and age-adjusted criteria in NP, and between strict criteria and loose criteria in dBC. As shown in Appendix Figure S1, Supplemental Digital Content, https://links.lww.com/MD/O779, the age-adjusted criteria for NP could be used helpfully in studies in which the diagnosis of NP itself is important because it corrects well for the prevalence according to age. However, it is considered more efficient to use the absolute criteria since it is more important to evaluate the increase in NUV than to diagnose NP in determining the treatment of antidiuretics. In addition, as shown in the dBC, even if the same absolute criteria were applied, it became difficult to sufficiently express the change in the MVV when the criteria were strictly applied (Appendix Fig. S2, Supplemental Digital Content, https://links.lww.com/MD/O780). Therefore, when nocturia was analyzed using a mathematical calculation, there was a difference depending on the definition of the pathophysiology even if the same FVC was analyzed.

Then, how should FVC, which is so important for the diagnosis of nocturia, be utilized? The most important method is not to use a mathematical calculation, but to understand the FVC as a whole. Because nocturia has inter-day and inter-nocturia variations, the cause of each nocturia should be identified individually, rather than using the average value of the FVC for 1 day or 3 days. To do this, it is very important to evaluate whether the nocturia occurs with a full bladder. The evaluation method is described in the “study definition” of “materials and methods” of this paper. It is also important to assess the dBC using only day time voided volumes. In addition, the start and end times of sleep should be checked to determine the quality of sleep, which is closely related to the nocturia. Based on this, it is necessary to decide when to use antidiuretics, the necessity and method of correcting sleep disorders, and the method of behavioral therapy. These are very important factors when treating nocturia, but they are factors that cannot be identified at all using mathematical calculations simply.

This study had several drawbacks. First, there is a possibility of selection bias because it is a retrospective study. This can act as an important bias because the causes of hospital visits could differ according to age. Second, the severity of nocturia was lower than that of other studies because this study was conducted on patients who complained of nocturia among patients with lower urinary tract symptoms.

In conclusion, the mathematical calculation used to analyze the pathophysiology of nocturia using an FVC has several problems such as differences according to the duration of FVC, inability to express inter-day and inter-nocturia variations, and differences according to diagnostic criteria for pathophysiology. Therefore, this mathematical calculation is not helpful when determining the treatment modality for nocturia. A more appropriate method for FVC analysis is needed to understand the FVC as a whole for treatment of nocturia.

## Author contributions

**Conceptualization:** Seong Cheol Kim.

**Data curation:** Woocheol Kang, Seong Cheol Kim.

**Formal analysis:** Woocheol Kang, Eun Ji Park, Seong Cheol Kim.

**Investigation:** Ji Hyung Yoon, Taekmin Kwon, Seong Cheol Kim.

**Methodology:** Seong Cheol Kim.

**Project administration:** Seong Cheol Kim.

**Resources:** Hoyoung Bae, Ji Hyung Yoon, Taekmin Kwon, Sungchan Park, Kyung Hyun Moon, Sang Hyeon Cheon.

**Supervision:** Dong-Gi Lee, Sungchan Park, Kyung Hyun Moon.

**Validation:** Sungchan Park, Sang Hyeon Cheon.

**Visualization:** Woocheol Kang, Dong-Gi Lee, Seong Cheol Kim.

**Writing – original draft:** Seong Cheol Kim.

**Writing – review & editing:** Dong-Gi Lee, Sungchan Park, Seong Cheol Kim.

## Supplementary Material


