# Supplementary material for: Is it effective to do mathematical analysis for the etiology of nocturia using the nocturia indices derived from the frequency volume chart?: A retrospective observational study
Source: Medicine (Baltimore). 2025 May 9;104(19):e42222. doi: 10.1097/MD.0000000000042222 (PMC12074110; doi:10.1097/MD.0000000000042222)
Supplement: Supplementary file 1 [file medi-104-e42222-s001.docx]

**Supplementary appendix table 1.** Difference between 1 day and ≥3 days

| **Duration of FVC** | **1 day** | **≥3 days** | **p-value** |
| --- | --- | --- | --- |
| MVV (mL) |  |  | <0.001 |
| Median | 330 | 400 |  |
| IQR | 300-450 | 300-500 |  |
| dBC (%) | 49.8 | 31.9 | <0.001 |
| dNBC (%) | 29.0 | 45.3 | <0.001 |

dBC, decreased bladder capacity; dNBC, decreased nocturnal bladder capacity; FVC, frequency volume chart; IQR, interquartile range; MVV, maximal voided volume
