# Supplementary material for: Is it effective to do mathematical analysis for the etiology of nocturia using the nocturia indices derived from the frequency volume chart?: A retrospective observational study
Source: Medicine (Baltimore). 2025 May 9;104(19):e42222. doi: 10.1097/MD.0000000000042222 (PMC12074110; doi:10.1097/MD.0000000000042222)

**Supplementary appendix figure 1.** Serial change in nocturnal polyuria index according to age. The black solid line is definition 1 (NPi>0.35), and the red dot line is definition 2 (NPi0.20-0.33). Definition 1 reflects the increase in NP with age, but definition 2 keeps the diagnosis of the nocturnal polyuria constant regardless of age; **NPi**=nocturnal polyuria index


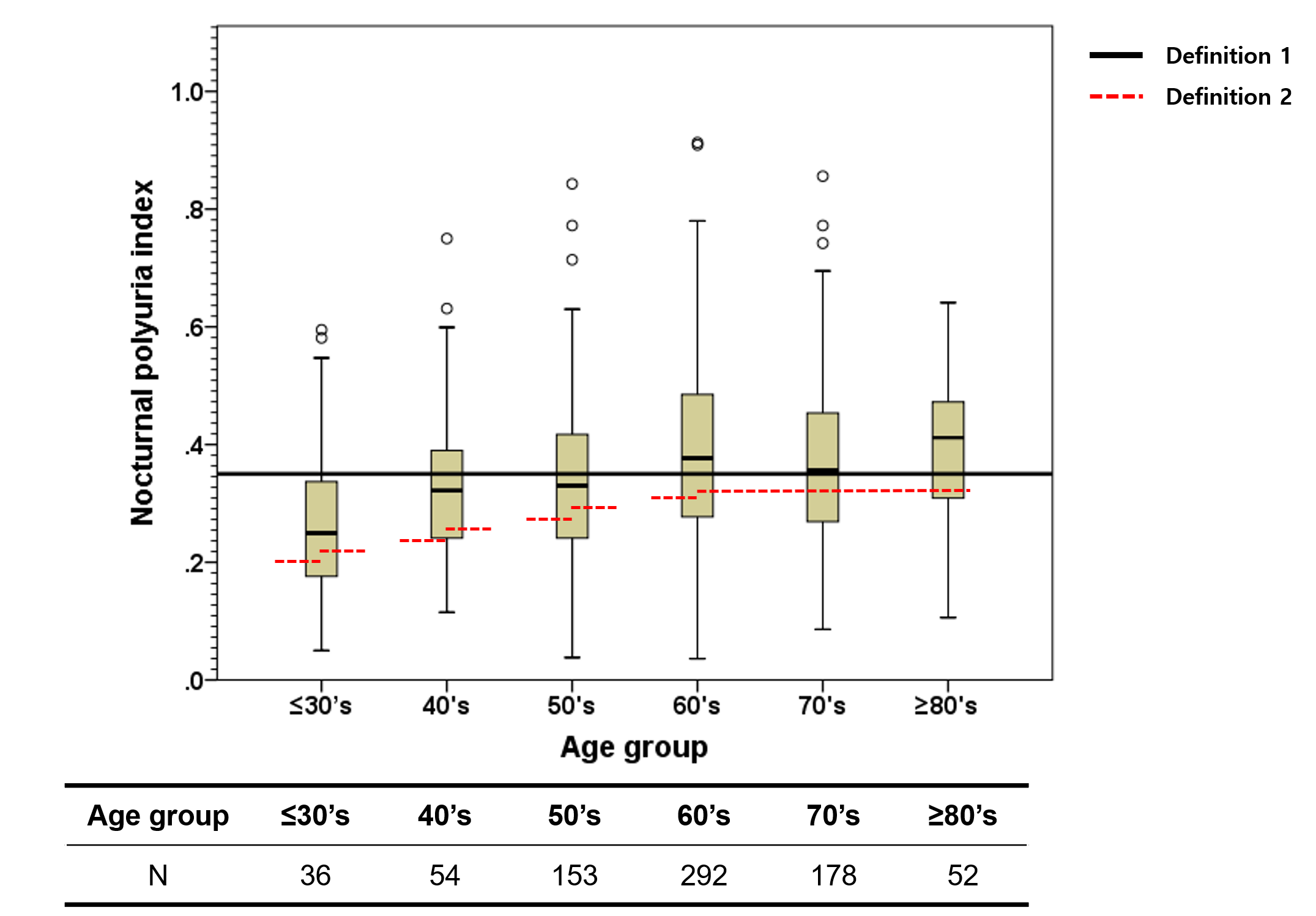

Supplement: Supplementary file 2 [file medi-104-e42222-s002.docx]
