# Supplementary material for: Is it effective to do mathematical analysis for the etiology of nocturia using the nocturia indices derived from the frequency volume chart?: A retrospective observational study
Source: Medicine (Baltimore). 2025 May 9;104(19):e42222. doi: 10.1097/MD.0000000000042222 (PMC12074110; doi:10.1097/MD.0000000000042222)

**Supplementary appendix figure 2.** Serial change in maximal voided volume according to (a) the number of nocturia episodes and (b) age. The black solid line is definition 1 (MVV<325 mL), and the blue dot line is definition 2 (MVV<200 mL). The two graphs show that definition 2 is unable to reflect the serial change of MVV according to the number of nocturia episodes and age; **MVV**=maximal voided volume


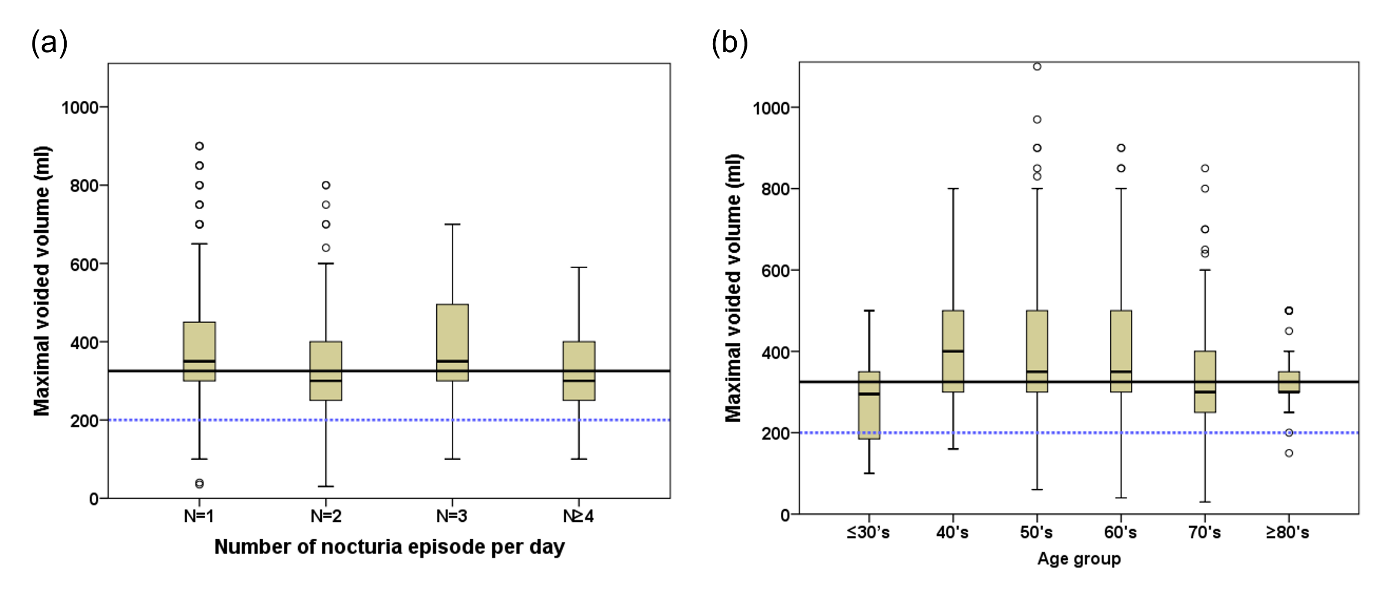

Supplement: Supplementary file 3 [file medi-104-e42222-s003.docx]
